# Contrasting epigenetic states of heterochromatin in the different types of mouse pluripotent stem cells

**DOI:** 10.1038/s41598-018-23822-4

**Published:** 2018-04-10

**Authors:** Matteo Tosolini, Vincent Brochard, Pierre Adenot, Martine Chebrout, Giacomo Grillo, Violette Navia, Nathalie Beaujean, Claire Francastel, Amélie Bonnet-Garnier, Alice Jouneau

**Affiliations:** 1 UMR BDR, INRA, ENVA, Université Paris Saclay, 78350 Jouy en Josas, France; 2 UMR7216 Epigenetics and cell fate, Université Paris Diderot Paris 7, 75013 Paris, France; 3 Present Address: Univ Lyon, Université Claude Bernard Lyon 1, Inserm, INRA, Stem Cell and Brain Research Institute U1208, USC1361, 69500 Bron, France

## Abstract

Mouse embryonic stem cells (ESCs) and epiblast stem cells (EpiSCs) represent naive and primed pluripotency states, respectively, and are maintained *in vitro* by specific signalling pathways. Furthermore, ESCs cultured in serum-free medium with two kinase inhibitors (2i-ESCs) are thought to be the ground naïve pluripotent state. Here, we present a comparative study of the epigenetic and transcriptional states of pericentromeric heterochromatin satellite sequences found in these pluripotent states. We show that 2i-ESCs are distinguished from other pluripotent cells by a prominent enrichment in H3K27me3 and low levels of DNA methylation at pericentromeric heterochromatin. In contrast, serum-containing ESCs exhibit higher levels of major satellite repeat transcription, which is lower in 2i-ESCs and even more repressed in primed EpiSCs. Removal of either DNA methylation or H3K9me3 at PCH in 2i-ESCs leads to enhanced deposition of H3K27me3 with few changes in satellite transcript levels. In contrast, their removal in EpiSCs does not lead to deposition of H3K27me3 but rather removes transcriptional repression. Altogether, our data show that the epigenetic state of PCH is modified during transition from naive to primed pluripotency states towards a more repressive state, which tightly represses the transcription of satellite repeats.

## Introduction

Pluripotency is defined as the ability of a stem cell to generate all three embryonic lineages. In the mouse, culture conditions have allowed capture of different pluripotency states *in vitro*, which leads to distinguish a naive from a primed state of pluripotency^1^. These two states functionally differ in their ability to produce chimaeras. Naive embryonic stem cells (ESCs) integrate into the inner cell mass (ICM) upon injection into a blastocyst and subsequently contribute to all tissues of the chimaera. In contrast, primed epiblast stem cells (EpiSCs) cannot integrate into the ICM^2,3^; however, they form chimaeras with the post-implantation epiblast^4^. ESCs are classically maintained in serum-containing medium supplemented with LIF that activates the STAT3 signalling pathway but can also be cultured in serum-free medium with only inhibitors of two differentiation pathways: MAPK/ERK (mitogen-activated protein kinases/extracellular signal-regulated kinases) and GSK3 (glycogen synthase kinase 3) pathways^5^. In this 2i medium, cells are now in a ground naive state, with more efficient repression of lineage commitment markers and more homogenous expression of pluripotency genes than those cultured in serum/LIF^6,7^. EpiSCs are maintained using FGF and Activin A signalling pathways. Their transcriptome reflects their primed nature, as they already express many lineage commitment markers with concomitant down-regulation of some pluripotency genes^2,8^. ESCs cultured in serum/LIF (serum-ESCs) display an intermediate transcriptome, with heterogeneous levels of pluripotency markers at the single cell level and low but detectable expression of differentiation genes^6,9^.

ESCs are considered to have more open and plastic chromatin than differentiated cells^10,11^ with reduced nucleosome density^12^. Moreover, the epigenomic profiles of ESCs grown in 2i- or serum-containing medium is distinct but interconvertible upon exchange of culture medium^6,13,14^. Specifically, 2i-ESCs exhibit significantly reduced H3K27me3 deposition at promoters and global DNA hypomethylation^6,14^. ESCs can be converted into EpiSCs (cEpiSCs) *in vitro* when exposed to FGF and Activin signalling^15^. Although few in-depth analyses have been reported, available data indicate that during conversion to the primed state, many promoters become hypermethylated with substantial rearrangement of enhancer chromatin patterns compared to ESCs^16,17^. On the other hand, reverting EpiSCs into naive cells is a long and inefficient process, eliciting the notion of epigenetic barrier to reprogramming^18^.

These studies all suggest that each pluripotent cell type is characterized by a specific chromatin organization and epigenome. However, these comparisons have yet to be performed with regard to the constitutive heterochromatin compartment. This compartment forms at telomeres and pericentromeric regions, which are mainly composed of tandem repeats (reviewed in^19^), and proper control of these regions is crucial for chromosomal stability^20^.

In addition to telomeric sequences, there are two types of tandem repeats in the mouse genome: major satellite repeats at pericentromeric heterochromatin (PCH) and minor satellite repeats at centromeres^21^. The major satellite repeats consist of 234bp AT-rich sequences repeated over 200,000 times in tandem that represent approximately 3% of the mouse genome. During interphase in somatic cells, PCH from different chromosomes aggregates in clusters termed chromocenters that are typically enriched in the repressive SUV39H1/2-mediated histone mark H3K9me3^22^. DNA methylation is another hallmark of constitutive heterochromatin that coexists with H3K9me3 in substantial quantities^20^. Methylation of cytosine nucleotides (5-meC) is accomplished by DNA methyltransferases DNMT3A/B and maintained throughout cell divisions by DNMT1^23,24^. Despite this repressive epigenetic state, satellite transcripts can be transcribed and indeed participate in stabilization of the PCH structure^25,26^. However, increased transcript accumulation typically occurs in response to stress and some cancers, usually coincident with reduced DNA methylation at these sequences (reviewed in^27^). In serum-ESCs, although major satellites are also enriched in H3K9me3 and 5-meC^22,23^, transcription occurs at higher levels than in differentiated cells, such as neural progenitors^28^. PCH in serum-ESCs shows some plasticity, as demonstrated in mutants that lacks either H3K9me3 (*Suv39h* dn knockout) or DNA methylation (*Dnmt* knockout), where these missing marks are replaced by the typical facultative heterochromatin mark H3K27me3^22,29,30^.

As mentioned above, when ESCs are switched from serum-containing to 2i-containing medium, H3K27me3 is redistributed, reducing global DNA methylation across the genome^14^, suggesting that PCH organization may also be impacted. In addition, it is not known if organization of PCH and its transcription status are conserved in the primed EpiSCs. To address these questions, we performed a comparative study of the epigenetic and transcriptional landscapes of PCH sequences in naive and primed states of pluripotency. We show that PCH in naive 2i-ESCs exhibits unusual epigenetic marks, with a strong enrichment in H3K27me3, at the expense of both H3K9me3 and DNA methylation. In contrast, PCH in primed EpiSCs is characterized by somatic-like features, with dense deposition of H3K9me3 and 5-meC and repressed transcription of major satellite repeats. We also show that silencing of major satellites is largely independent of the presence of DNA methylation and H3K9me3 in ground naive ESCs whereas both marks are required in EpiSCs.

## Results

### Epigenetic landscapes at the pericentromeric heterochromatin in different pluripotent cells

**Figure 1:**
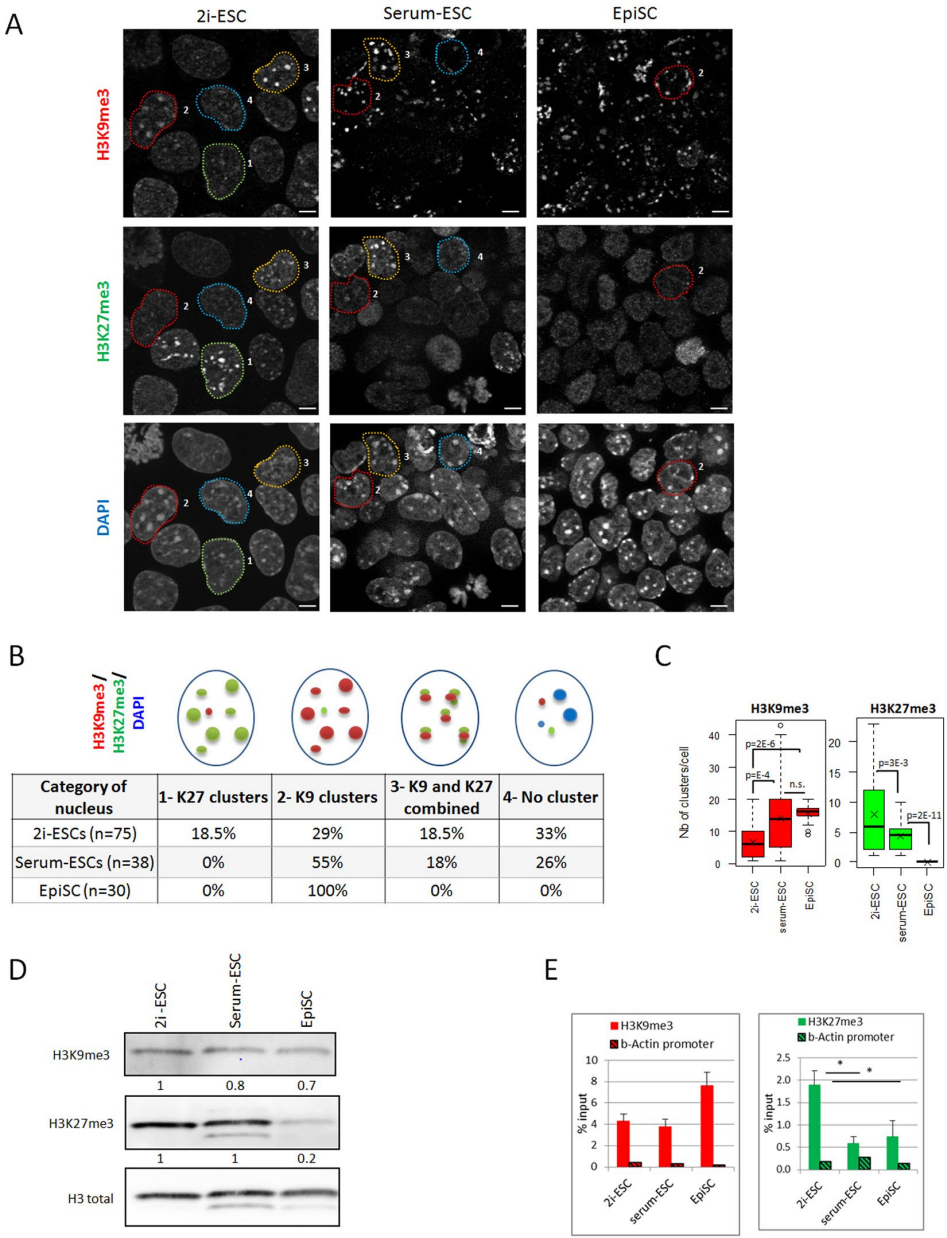
Heterochromatin landscape in the different states of mouse pluripotency (**A**) Single plan images of the 3 cell types immunolabeled with antibodies against H3K9 tri-methylation (H3k9me3) and H3K27 tri-methylation (H3K27me3). DNA is counterstained with DAPI. Scale bars represent 5µm. Encircled nuclei correspond to each of the 4 categories sketched in B. (**B**) Schematic drawings of the 4 categories of nuclei observed within the 2i-ESC population and their proportion in the 3 cell types. The number of cells analysed is indicated in the first column. (**C**) Boxplots showing the number of H3K9me3 and H3K27me3 clusters in positive nuclei. Pvalues were calculated using Student t-test; n.s. = not significant. (**D**) Western-blot analysis of H3K27me3 and H3K9me3 with H3 total as loading control in the 3 cell types. (**E**) ChIP-qPCR analysis of H3K9me3 and H3K27me3 at major satellites and beta-Actin promoter (to measure background level). Stars indicate statistically significant data and error bars are s.e.m (n = 4, Mann-Whitney U test, P value ≤ 5%).

We first examined the distribution of H3K9me3, the hallmark of heterochromatin, at PCH/CH regions in ESCs in serum and 2i conditions and in EpiSCs by immunofluorescence. As H3K27me3 can substitute for H3K9me3 at PCH^22,29,30^, we analysed both histone marks in the same cells (Fig. 1A). DAPI, which binds strongly to A-T rich regions, was used to label DNA and to distinguish the chromocenters that form dense clusters in interphase nuclei.

Strikingly, some nuclei exhibited dense H3K27me3 signal in chromocenters, specifically in 2i-ESCs. In addition, we observed great heterogeneity in labelling patterns for both types of ESCs (serum and 2i). To compare the different cell types, we defined four nuclei categories (schematic drawings in Fig. 1B): category 1: nuclei with a majority (and at least 4) of chromocenters labelled with H3K27me3; category 2: nuclei with a majority (and at least 4) of chromocenters labelled with H3K9me3; category 3: nuclei with a majority (and at least 4) of chromocenters labelled with both repressive marks (closed to each other or overlapping) and category 4: nuclei with few labelled chromocenters or chromocenters without any repressive histone marks. Representative nuclei for each category are shown in Supplementary Fig. 1A. We then assessed the percentage of nuclei in each category for the three cell types (Fig. 1B) and quantified the number of DAPI, H3K27me3 and H3K9me3 clusters *per* nucleus (category 1–3) for each cell type (Fig. 1C, Table in Supplementary Fig. 1).

Distribution of the four categories was statistically different between the two types of ESCs (Chi-squared test, p = 10–4). Only 2i-ESCs fell into the category 1 (18.5%) and the mean number of H3K27me3 clusters in all H3K27me3 positive cells (sum of categories 1 and 3) was higher in 2i- than in serum-ESCs (n = 8 vs n = 4; Fig. 1C). The percentage nuclei in category 2 was higher in serum- than in 2i-ESCs (55% vs 29%) as well as the mean number of H3K9me3-enriched chromocenters (n = 14 in serum-ESCs vs n = 7 in 2i-ESCs; Fig. 1C). Category 3 was equally represented in both types of ESCs (18–18.5%; Fig. 1B). Clusters lacking repressive marks were observed in 26% and 33% of ESCs in serum- and 2i-medium, respectively (category 4, Fig. 1B).

In primed EpiSCs, no such heterogeneity was observed since all nuclei showed only H3K9me3 clusters perfectly co-localizing with chromocenters (100% category 2, Fig. 1B and Supplementary Fig. 1A), along with faint and diffuse staining for H3K27me3. The mean number of H3K9me3 clusters also significantly increased in EpiSCs compared to serum-ESCs (Fig. 1C). This contrasting pattern between EpiSCs and ESCs prompted us to quantify global levels of both histone modifications by western-blotting (Fig. 1D). We thereby confirmed a substantial loss of H3K27me3 in EpiSCs compared to both ESCs and a steady level of H3K9me3.

Finally, to confirm the enrichment of either H3K9me3 or H3K27me3 in PCH, we used ChIP-qPCR assays (Fig. 1E). We confirmed a significant enrichment of H3K27me3 in 2i-ESCs compared to serum-ESCs and EpiSCs at major satellite sequences. Conversely, H3K9me3 tended to be more enriched in EpiSCs at major satellites, although these results did not reach statistical significance.

**Figure 2:**
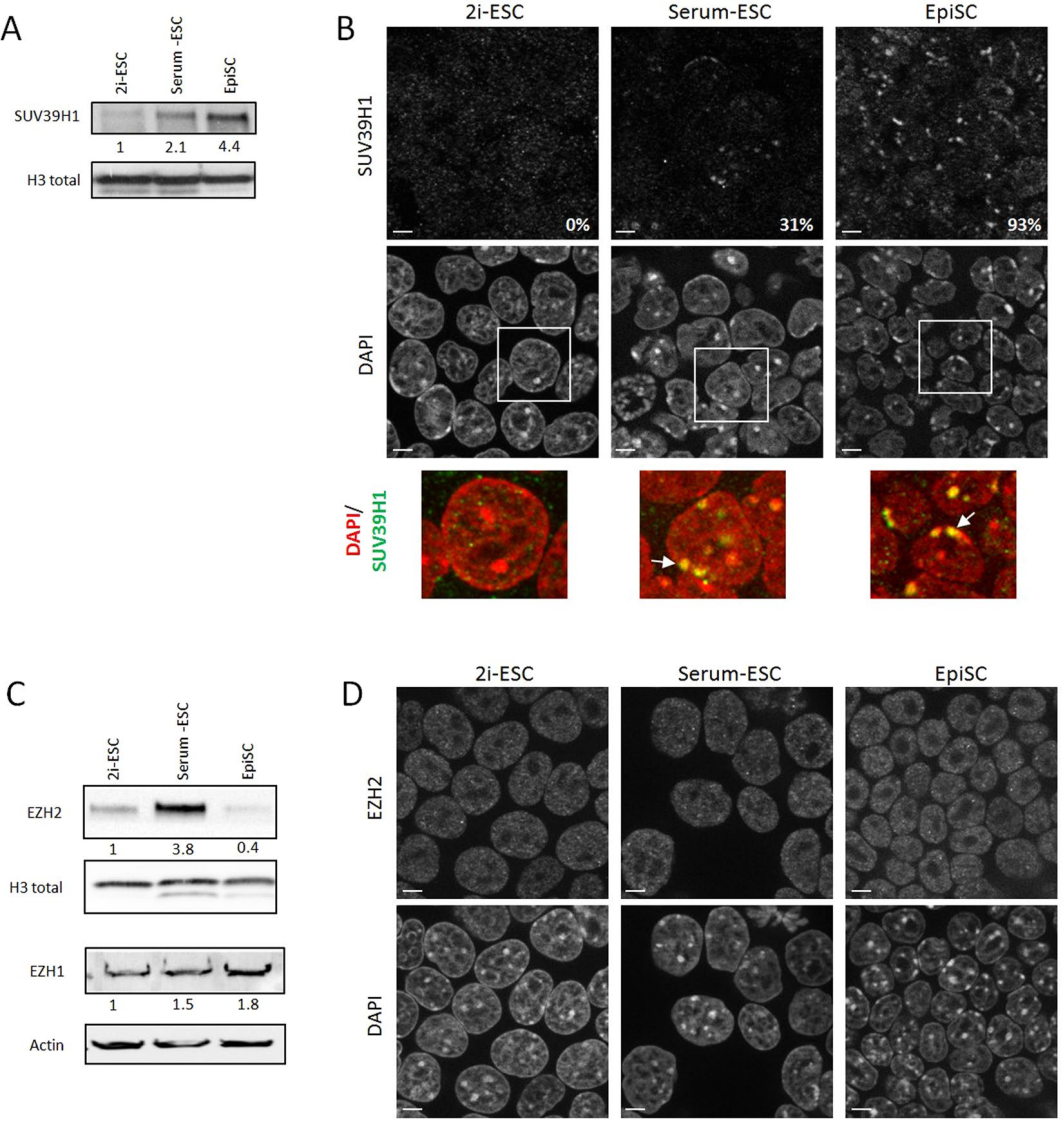
Expression and pattern of epigenetic modifiers (**A**) Western-blots for SUV39H1 and H3. (**B**) Single plan images of the 3 cell types immunolabelled with antibody against SUV39H1 (scale bars represent 5 µm). A representative enlarged nuclei (white squares, lower panel, DNA in red and SUV39H1 in green) and the percentage of cells in the population displaying DAPI clusters enriched with SUV39H1 (white arrows) are indicated for each cell type. (**C**) Western-blots for PRC2 enzymes, EZH2 and EZH1, and H3 or Actin. (**D**) Single plan images of the 3 cell types immunolabelled with antibody against EZH2 (scale bars represent 5 µm).

As the SUV39H enzymes catalyse the methylation of H3K9 at PCH^31,32^ and EZH1/2, part of the PRC2 complex, deposits H3K27me2/3^33^, we next examined the protein levels of these enzymes in the three pluripotent cell types (Fig. 2). SUV39H1 levels correlated well with increased levels of H3K9me3 at PCH in primed compared to naive pluripotent cells (Fig. 2A). Of note, immunostaining revealed diffuse distribution in 2i-fed ESCs, while 31% of the serum-exposed ESCs and the vast majority (93%) of EpiSCs exhibited SUV39H1 accumulation in DAPI-dense clusters (Fig. 2B). On the other hand, protein levels of EZH2 also varied within the different cell types, but in the opposing pattern: EZH2 was reduced in EpiSCs relative to ESCs whereas the highest levels were detected in serum-ESCs (Fig. 2C). However, the distribution of EZH2 immunostaining was quite similar regardless of cell type, as we observed the same punctuated nuclear signal with occasional bright, tiny spots (Fig. 2D). EZH1 expression was low with no obvious change between ESCs and EpiSCs (Fig. 2C).

Altogether, our data suggest that H3K27me3 distribution at PCH discriminates the naive ground state from other pluripotent cells. This distinct pattern is lost upon acquisition of the primed state, replaced by a uniform enrichment of H3K9me3 at chromocenters.

### Low levels of DNA methylation at PCH in 2i-ESCs correlate with low levels of DNMT3A/B enzymes

**Figure 3:**
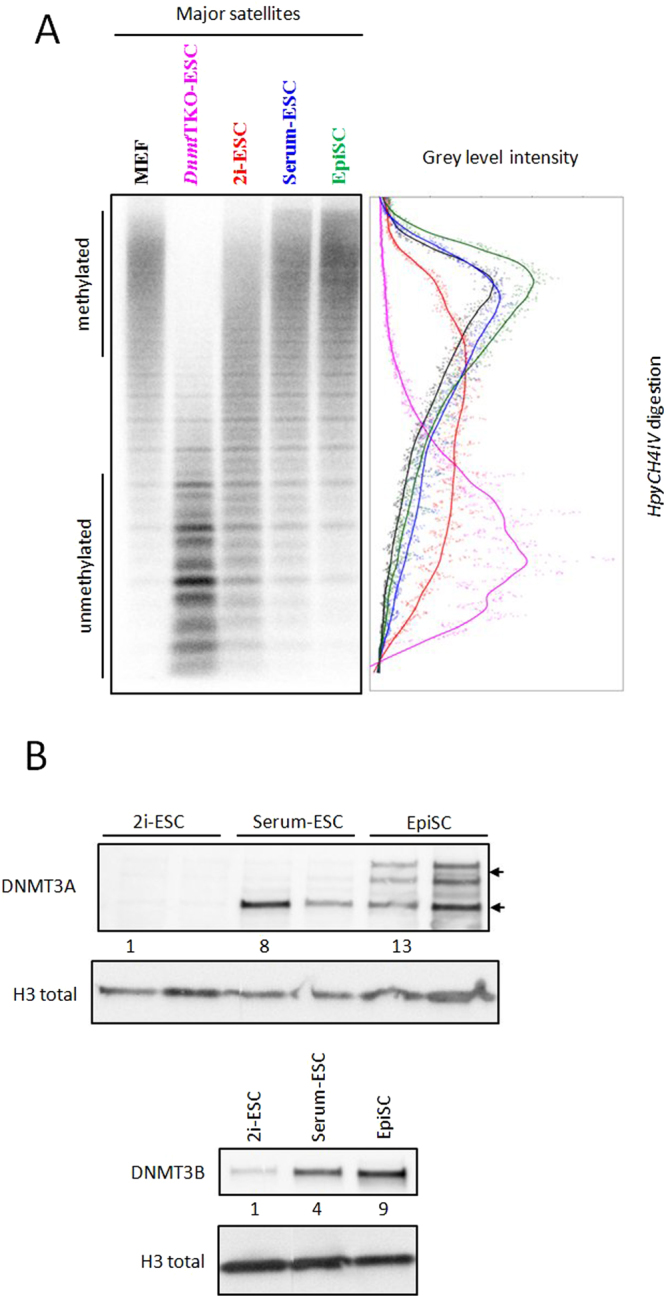
Methylation profile at major satellites. (**A**) Southern-blot analysis of gDNA digested with *HpyCH4IV* revealed with probe for major satellites. Linescan quantification for each lane: MEF (black), *Dnmt*TKO (pink), 2i-ESC (red), serum-ESC (blue) and EpiSC (green). (**B**) Western-blots for DNMT3A, DNMT3B and H3.

To further dissect the status of PCH in each subset of pluripotent cells, we examined DNA methylation levels at major satellite sequences by Southern-blotting after genomic digestion with methylation-sensitive enzymes. We observed dramatic differences at these sequences between the different pluripotent states (Fig. 3A). Major satellites were partially demethylated in 2i-ESCs, as shown by the line scan profile (red line), which is between fully demethylated *Dnmt*TKO cells (purple line) and methylated somatic cells (MEFs - black line) (Fig. 3A). In contrast, levels of DNA methylation at these sequences in both serum-ESCs (blue line) and EpiSCs (green line) were comparable to those found in MEFs.

We next compared expression levels of *de novo* DNA methylation enzymes by western-blotting among the different pluripotent states (Fig. 3B). *Dnmt3a* produces two different isoforms, the lower form being predominant in embryonic tissues, while the upper form is present in somatic cells^34^. DNMT3A was nearly undetectable in 2i-ESCs, serum-ESCs gained the embryonic form, and both isoforms were detected in EpiSCs (Fig. 3B). DNMT3B protein levels exhibited incremental increases 2i- to serum-ESCs and finally EpiSCs (Fig. 3B).

In conclusion, we observed progressive methylation of satellite sequences, accompanied by an increased expression of the *de novo* methyltransferases in 2i-supplemented ESCs compared to EpiSCs.

### PCH transcription levels are highly variable depending on the state of pluripotency

**Figure 4:**
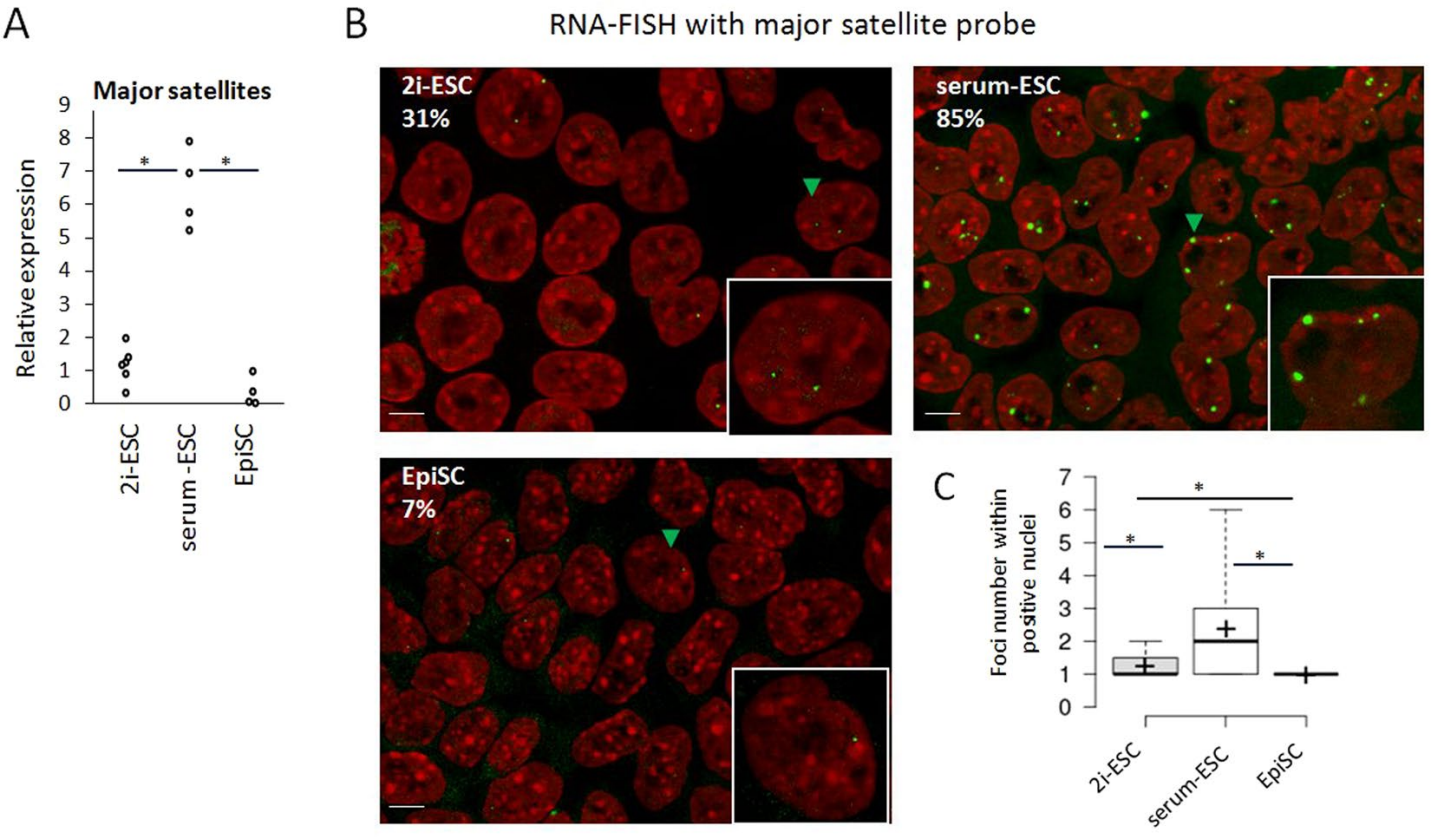
Transcription of major satellites (**A**) Relative expression (CNRQ) of major satellite transcripts by qRT-PCR analysis normalized to *Sdha* and *Pbgd* housekeeping genes. Each point is an independent biological replicate. Statistically significant data are indicated by an asterisk (P < 5%, Mann-Whitney U test). (**B**) Detection of major satellite transcripts using RNA-FISH. Transcripts localization appears as green foci while DNA is counterstained with DAPI (red). Percentage of positive nuclei is indicated for each cell type as well as an enlarged representative nucleus (indicated in the cell field by a green arrowhead). Scale bars represent 5 µm. (**C**) Boxplots showing the number of foci/ positive nucleus. Mean is indicated by a cross. Statistically significant data are indicated by an asterisk (P < 0.3%, Student test).

Next, we evaluated transcript levels of major sequences by RT-qPCR in ESCs and EpiSCs (Fig. 4A). Surprisingly, major satellite transcripts were present at lower levels in 2i-ESCs than in serum-ESCs. In primed EpiSCs, expression was even lower than in 2i-ESCs. To confirm this observation, we performed RNA-FISH using probes against major satellites (Fig. 4B). Discrete foci within the nucleus revealed accumulation of transcripts in ESCs. They were found next to small chromocenters, or within the pre-nuclear or peri-nucleolar heterochromatin (see examples in Supplementary fig. 2). As expected, serum-ESCs exhibited a high proportion of positive cells (85%) with prominent accumulation of major satellite transcripts (1–6 foci *per* cell; Fig. 4C). In contrast, few foci were found in 2i-ESC nuclei (~1 foci in 31% of the cells) and almost none were observed in EpiSC (7% of the cells).

In conclusion, we observed considerable changes in the transcription of satellite sequences based on the state of pluripotency (ESCs vs EpiSCs) and the culture medium of ESCs (2i vs serum). The abundancy of satellite transcripts in serum-ESCs compared to 2i-ESCs or EpiSCs suggests that such abundance is not linked to the state of pluripotency but to the presence of yet unknown transcription factors induced by serum components.

In light of this data, we decided to focus on the two extreme states of pluripotency, the ground naive 2i-ESCs and the primed EpiSCs, both cultured in serum-free medium but maintained by induction of different signalling pathways and exhibiting different features at PCH. Using these two models, we investigated the control exerted by epigenetic marks on levels of satellite transcripts.

### Reduced levels of H3K27me3 do not significantly affect levels of major satellite sequence transcripts

**Figure 5:**
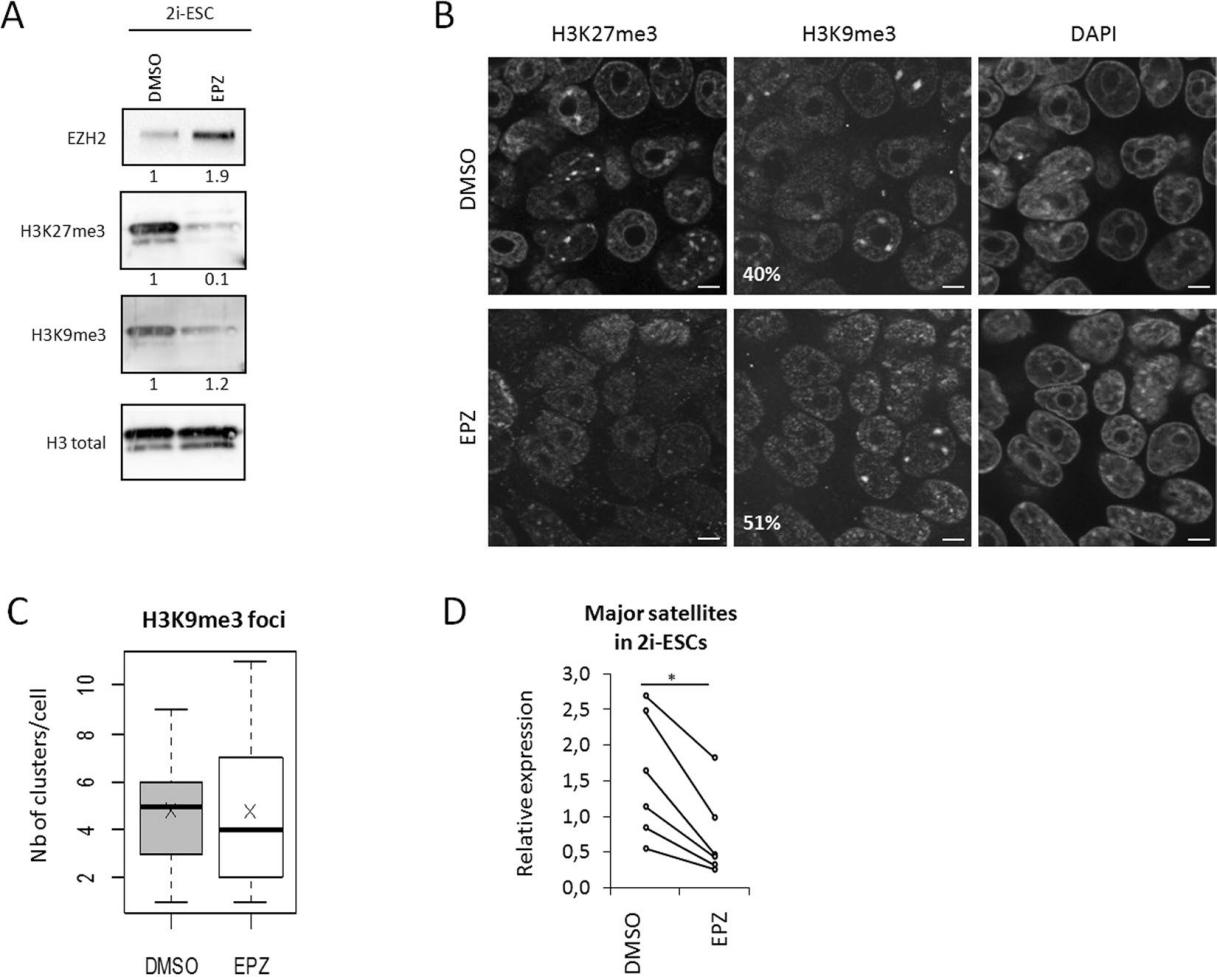
Inhibition of H3K27me3 in 2i-ESCs. (**A**) Western-blot analysis of EZH2, H3K9me3 and H3K27me3 related to total H3 in 2i-ESC treated with DMSO (Control) or EPZ (EZH2 inhibition). (**B**) Immunostaining images (single-plan) for H3K9me3 and H3K27me3 with DAPI DNA counterstaining in 2i-ESC treated with DMSO or EPZ. Percentages of cells presenting H3K9me3 clusters are indicated. Scale bars represent 5 µm. (**C**) Boxplots showing the number of H3K9me3 clusters in 2i-ESC treated with DMSO or EPZ. p-values were calculated using Student t-test; n.s. = not significant. (**D**) Relative expression (CNRQ) of major satellites transcripts by qRT-PCR analysis normalized to *Sdha* and *Pbgd* housekeeping genes in 2i-ESC treated with DMSO or EPZ. Each point is an independent biological replicate. Statistically significant data are indicated by an asterisk (P < 5%, Mann-Whitney U test for paired data).

We first investigated the role of H3K27me3 in the regulation of PCH transcriptional status. To that end, we used an inhibitor of the methyl-transferase activity of EZH2, EPZ-6438 (EPZ)^35,36^. Treatment of ESCs with EPZ for 72 hours led to a massive reduction in bulk levels of H3K27me3 (at least 70% in each cell type tested), with no change in H3K9me3 levels (Fig. 5A). Additionally, we observed that EZH2 levels were increased in EPZ treated cells (Fig. 5A). Inhibition of EZH2 enzymatic activity may lead to reduced turnover of this protein and hence its accumulation. Loss of H3K27me3 clusters in 2i-exposed ESCs treated with EPZ was also confirmed by immunostaining (Fig. 5B). In addition, the proportion of cells with H3K9me3 clusters at chromocenters in DMSO or EPZ treated cells was not significantly affected (40% vs 51%; Fig. 5B) and the mean number of clusters *per* nucleus remained unchanged (n = 5 vs 7; Fig. 5C). Finally, the level of DNA methylation at major satellite repeats was also unaffected in response to EPZ treatment (Data not shown). Hence upon EPZ treatment, only the enrichment of H3K27me3 at PCH was affected. We then assessed the levels of major satellite transcripts in this context in 2i-ESCs and observed a slight but significant reduction in PCH transcripts levels (Fig. 5D).

Consequently, reduction of H3K27me3 at PCH in naive ESCs does not induce up-regulation of major satellite transcripts.

### The absence of *Suv39h1/2* derepresses satellite transcription in primed pluripotent cells

We next examined the role of H3K9me3 on the regulation of satellite transcript levels using *Suv39h*dn mutant ESCs, in which both Suv39h enzymes are knocked-out^22^. We adapted these ESCs to 2i culture conditions and converted them *in vitro* into EpiSCs (cEpiSCs, Supplementary Fig. 3A). These cEpiSCs expressed the expected markers of primed pluripotency (Supplementary Fig. 3B) and could be stably propagated. These cells were immunostained for H3K9me3 and H3K27me3. Of note, corresponding wild-type cells (WT01 ESC line) displayed a higher proportion of nuclei enriched in H3K27me3 clusters and proportionally fewer nuclei with H3K9me3 clusters, compared to the R1 ESC line used above (Supplementary Fig. 2E compared to Fig. 1B).

**Figure 6:**
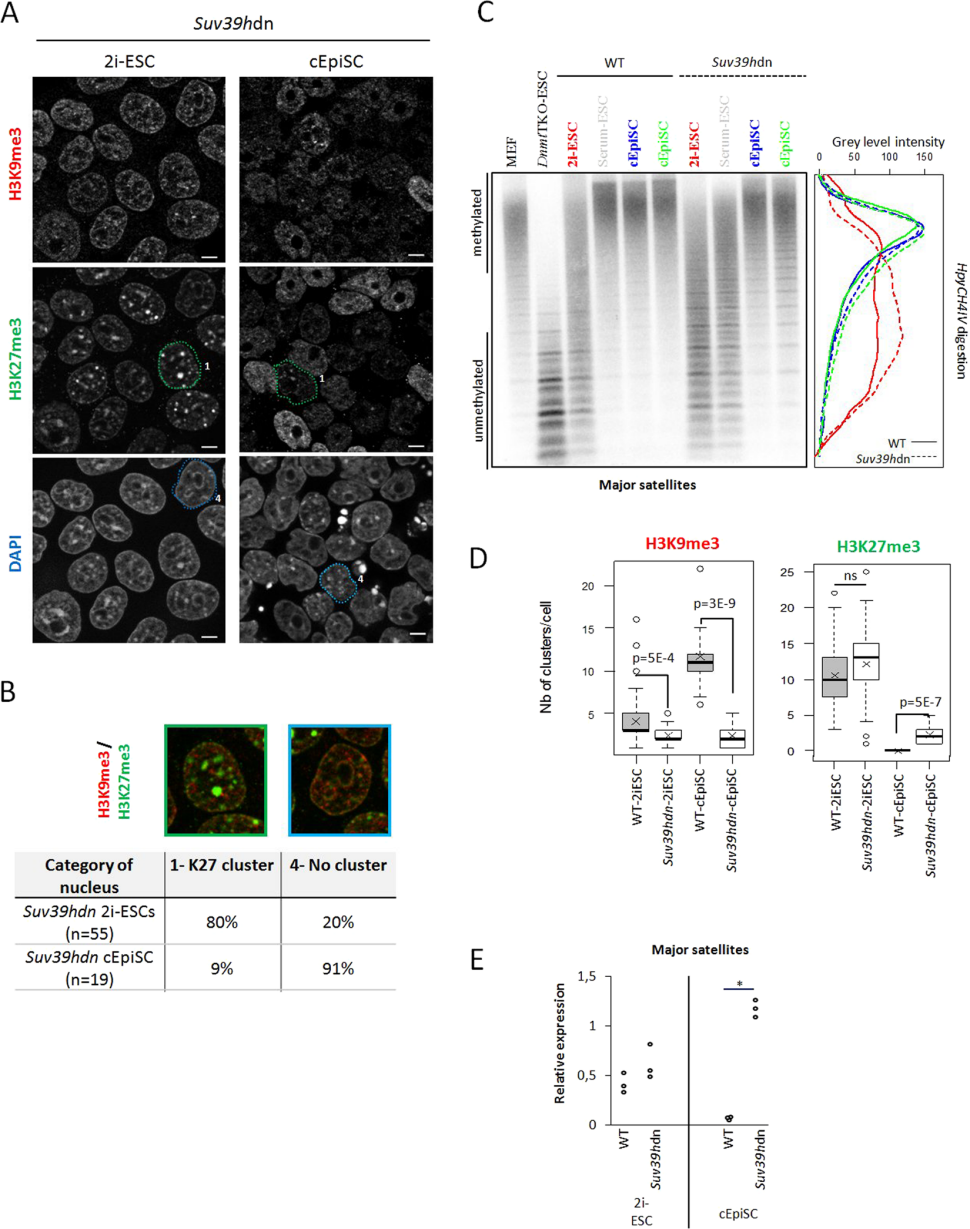
Organization at PCH and major satellite transcript level in *Suv39hdn* cells. (**A**) Immunostaining images (single-plan) for H3K9me3 and H3K27me3 with DAPI DNA counterstaining in *Suv39h*dn condition, to compare with wild-type condition (Supplementary Fig. 2D). Encircled nuclei are representative examples of category 1 and 4. Scale bars represent 5µm. (**B**) Representative nuclei of the two categories as defined in Fig. 1B and percentages of each in the *suv39hdn* 2i-ESC and cEpiSC populations. (**C**) Southern-blot analysis of gDNA digested with *HpyCH4IV* revealed with probe for major satellites in wild-type and *Suv39h*dn conditions. Linescan quantification for 2i-ESC (red) and EpiSC (blue and green). Wild-type condition is represented with a continuous line, while *Suv39h*dn with a dotted line. (**D**) Box-plots showing the number of clusters of H3K9me3 or H3K27me3 in positive cells in WT and mutant ESC and cEpiSC. Pvalues (student tests) are shown. (**E**) Relative expression (CNRQ) of major satellites transcripts by qRT-PCR analysis normalized to *Sdha* and *Pbgd* housekeeping genes in wild-type and *Suv39h*dn condition. Each point is an independent biological replicate. Statistically significant data are indicated by an asterisk (P < 5%, Mann-Whitney U test).

In *Suv39h*dn 2i-ESCs, H3K9me3 levels were globally reduced (western-blot, Supplementary Fig. 2C), more specifically at major satellites, as expected (ChIP-qPCR, Supplementary Fig. 3F). Surprisingly, 80% of mutant cell nuclei exhibited between 1 to 5 small H3K9me3 spots (mean = 2; Fig. 6D). We speculate that other histone methyltransferase (KMT) enzymes, such as SETDB1, may have deposited these small H3K9me3 spots. Previous data have indeed shown a reduction of H3K9me3, not only at euchromatin but also at PCH in the absence of SETDB1^37,38^. Among these spots, no more than two were localized close to chromocenters and always next to a larger H3K27me3 cluster. Hence, such nuclei were not counted as category 3. The majority (80%) of *Suv39h*dn 2i-ESCs were in category 1, the others being in category 4 (Fig. 6A,B). The number of H3K27me3 clusters *per* nucleus and its enrichment at major satellites were similar between WT and *Suv39h*dn ESCs in 2i culture conditions (Fig. 6D,F). In addition, the same low level of DNA methylation was observed at major satellites (Fig. 6C). We then compared the abundance of major satellite transcripts by RT-qPCR and found it unchanged in mutant compared to wild-type ESCs (Fig. 6D).

*Suv39h*dn cEpiSCs also displayed few small H3K9me3 spots (Fig. 6D), but in contrast to ESCs, they gained only a few H3K27me3 clusters and only in a minority of nuclei (9%; Fig. 6A,B,D). Thus, H3K27me3 was lost at major satellite sequences during conversion, as confirmed by ChIP-qPCR data (Supplementary Fig. 3F). In addition, DNA methylation was re-established at PCH in cEpiSCs and major satellites were similarly methylated in converted WT and mutant cells (Fig. 6C). Intriguingly, transcription of major satellites was de-repressed in *Suv39h*dn cEpiSCs, and transcript levels increased approximately ten-fold compared to WT cEpiSCs (Fig. 6E). Therefore H3K9me3 enrichment at PCH in primed EpiSCs prevents the accumulation of major satellites.

### The absence of DNA methylation increases deposition of H3K27me3 in ESCs and causes substantial accumulation of satellite transcripts in EpiSCs

Finally, we addressed the role of DNA methylation in the repression of PCH transcription. *Dnmt1*, *3a*, and *3b* triple knockout ESCs (*Dnmt*TKO) and their corresponding wild-type (WT) ESCs were adapted to 2i medium. These cells have no methylated cytosine in their genome^39^ and notably at PCH (see Fig. 3A). We also converted these cells into cEpiSCs (Supplementary Fig. 4A). *Dnmt*TKO and WT cEpiSCs expressed the expected markers of primed pluripotency with down regulated naive markers (Supplementary Fig. 4B). We were unable to maintain the converted mutant cells over numerous division cycles, in agreement with the reported impaired differentiation potential of these cells^40^.

**Figure 7:**
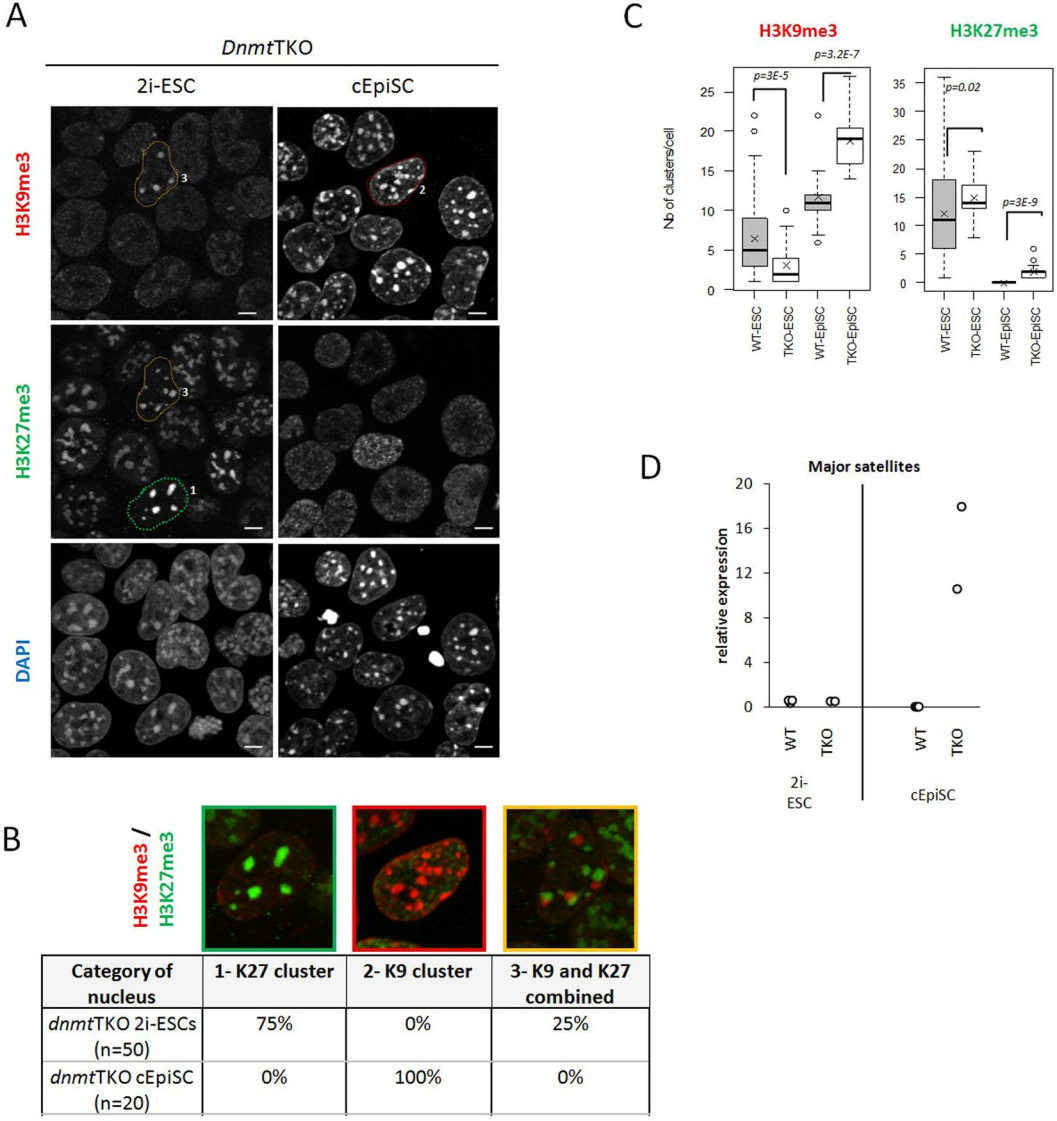
Organization at PCH and major satellite transcript level in *Dnmt*TKO cells. (**A**) Immunostaining images (single-plan) for H3K9me3 and H3K27me3 with DAPI DNA counterstaining in *Dnmt*TKO condition. Encircled nuclei are representative examples of categories 1, 2 and 3. Scale bars represent 5µm. (**B**) Representative nuclei of the categories as defined in Fig. 1B and percentages of each in the 2i-ESC and cEpiSC populations. (**C**) Box-plots showing the number of clusters of H3K27me3 in positive cells in WT and mutant ESC and cEpiSC. Pvalues (student tests) are shown. (**D**) Relative expression (CNRQ) of major satellites transcripts by qRT-PCR analysis normalized to *Sdha* and *Pbgd* housekeeping genes in Wild-type and *Dnmt*TKO condition.

In 2i-ESCs, the complete absence of DNA methylation is correlated with an increased proportion of cells with only H3K27me3 clusters (75% in 2i-ESCs *Dnmt*TKO vs 40% in corresponding WT; see Fig. 7B and Supplementary Fig. 4C). A slight increase in the mean number of clusters per nucleus was also observed (n = 12 in WT vs n = 14 in mutant ESCs; Fig. 7C). In addition, no cell with only H3K9me3 clusters (category 2) was observed in *Dnmt*TKO 2i-ESCs (Fig. 7B), and the mean number of clusters per nucleus was reduced (n = 6 in WT vs n = 3 in mutant ESCs; Fig. 7C).

In *Dnmt*TKO cEpiSCs, most H3K27me3 clusters disappeared, and all cells displayed H3K9me3 clusters (Fig. 7A,B). These clusters were even more numerous than in WT cEpiSC cells (n = 19 vs n = 12; Fig. 7C). A few nuclei displayed some small and faint H3K27me3 clusters (n = 2, Fig. 7C), but none of these colocalized with DAPI (Fig. 7B).

Interestingly, similar to the results obtained with *Suv39h*dn cells, the levels of major satellite transcripts were almost unchanged in mutant ESCs but dramatically increased in *Dnmt*TKO cEpiSCs compared to WT cells (Fig. 7C). This increase was more pronounced than in *Suv39h*dn primed cells, suggesting that the repressive effect of DNA methylation on transcription at PCH is stronger than that of H3K9me3.

## Discussion

Pluripotency is not a static state *in vivo* but rather a continuous process beginning in the ICM of the blastocyst and ending at the beginning of somitogenesis^41^. In the mouse, the different types of pluripotent cell lines that can be derived from embryos reflect this progression: 2i-ESCs, serum-ESCs and EpiSCs, the former being the closest to the ICM and the latter, the closest to the late epiblast. The PCH epigenetic state plays an important role in the maintenance of genome stability. Moreover, although satellite transcripts play a role in the formation of PCH^42,43^, their level must be tightly controlled^44^.

Herein, we performed a comprehensive comparison of the PCH epigenetic state in different pluripotent cells and showed that the regulation of transcription at PCH is substantially modified in response to the state of pluripotency: ground naive (2i) ESCs are characterized by strong enrichment of H3K27me3 at PCH and low levels of PCH satellite transcripts. In contrast, conversion to primed EpiSCs leads to considerable loss of this mark, prominent accumulation of DNA methylation and H3K9me3, as well as lower levels of PCH transcripts. Our second novel finding concerns repression of PCH transcription by DNA methylation and H3K9me3 only in primed EpiSCs, which we further demonstrate is independent of any classical repressive marks (H3K27me3, H3K9me3 and DNA methylation) in ground naive 2i- ESCs.

For all ESC cell lines used in our study, we observed H3K27me3 enrichment, which correlates with general demethylation at the DNA level^14^. Enrichment of H3K27me3 at PCH upon reduction of 5-meC has previously been observed in both *Suv39h*dn and in *Dnmt*TKO serum-ESCs^21,22,29,32^. Elsewhere in the genome, DNA methylation also antagonizes the deposition of H3K27me3. Indeed, in *Dnmt*TKO or 5-aza treated ESCs, H3K27me3 is redistributed to demethylated CpG sites at the expense of PRC2 canonical target sites^29,45^. Conversely, DNA methylation cannot substitute for H3K27me3, since the patterns of DNA methylation at promoters in PRC2 mutant ESCs are relatively unchanged^45^. Additionally, we did not observe evidence for an increase of 5meC at major satellites in 2i-ESCs treated with the EZH2 inhibitor. Upon switching ESCs from serum into 2i medium, considerable loss of H3K27me3 at gene promoters was observed^6^. Therefore, whereas this mark is reduced at unique sequences, we now show that it is redistributed to PCH in 2i-ESCs, making these cells an interesting model of the cross-talk between PRC regulation of transcription and PCH epigenetic state^20^.

Furthermore, we compared the level of major satellite transcripts in all three pluripotency states and found that transcription is high in serum-containing ESCs, reduced after transition to 2i medium, and even more repressed in the primed EpiSC state. The difference in transcript levels between serum- and 2i-ESCs cannot be explained by the epigenetic marks, as H3K9me3 is similarly globally enriched at PCH, and DNA methylation is reduced in 2i-ESCs compared to serum. In addition, our study now shows that none of the three repressive marks we examined exerts a direct repression on major satellite transcription in 2i-ESCs. Transcription of major satellite repeats is regulated during the cell cycle, with the greatest induction occurring during late G1 phase^25^. However, a recent study shows that upon adaptation of ESCs to 2i medium, G1 phase is prolonged, particularly the late G1 phase^46^. Despite this, such cell cycle differences do not correlate well with differences in major satellite transcripts. Hence, our data raise the question of the nature of the regulators (activators and repressors) of satellite transcription in ESCs. Indeed, satellite repeat sequences contain consensus binding sites for different transcription factors, among them Pax3 and Pax9^47^. However, these two repressive factors are not expressed in ESCs or EpiSCs (data not shown). Transcription in serum-ESCs may be regulated by unknown factors induced by serum components and not found in the chemically defined medium used in the 2i condition. On the other hand, the inhibitors of MAPK and GSK3 pathways may themselves repress satellite transcription, but this hypothesis remains to be tested. We also observed that satellite transcription repression is slightly reduced upon treatment with the EZH2 inhibitor EPZ. Such treatment was shown to prime ESCs towards endoderm^48^. Hence, changes in the pool of active regulators, while enabling endoderm priming, also repress satellite transcription.

In marked contrast, the regulation of major satellite transcript accumulation in primed EpiSCs is tightly controlled by both H3K9me3 and DNA methylation. In the absence of either mark, major satellites transcription is strongly enhanced, suggesting that these marks play a synergistic role in repression. Interestingly, we confirmed results from a previous study that demonstrated converted mutant cells could efficiently revert to the ESC state if they were grown in 2i+LIF medium (^40^and data not shown), illustrating that DNA methylation stabilizes the primed state, as it does during differentiation^40^. DNA methylation is also essential for the homeostasis of EpiSCs, since converted mutant cells cannot be extensively maintained in culture, due to extensive cell death. Interestingly, such a strict dependency on genomic methylation has also been observed in human primed ESCs, in which acute deletion of Dnmt1 leads to DNA damage and rapid cell death^49^. Whether PCH with altered epigenetic marks and increased satellite transcription plays a role in cell death still needs to be addressed. In contrast, *Suv39h*dn-converted EpiSCs were maintained in culture for several passages with no apparent lethality. However, whether genomic stability or differentiation ability are affected remains to be elucidated^50^.

Our study reveals new observations about the cross-talk between different repressive marks. In 2i-ESCs, increased H3K27me3 levels in chromocenters are accompanied by a reduced number of H3K9me3 clusters and a reduced expression of SUV39H1. In addition to its reduced protein level in 2i-ESCs, the poor retention at PCH of SUV39H1 can also be explained by the reduction of DNA methylation, as binding to MBD proteins contribute to the stabilization of SUV39H at PCH^51,52^. Of note, a recent work demonstrated that the MEK inhibitor (in the 2i medium) increases the protein level of JMJD2C, a H3K9 histone demethylase^53^, which may contribute to destabilization of SUV39H enzymes at PCH.

The primed pluripotency state presents a markedly different PCH organization. Similar to somatic cells, they show high levels of H3K9me3, 5-meC, and SUV39H1 at DAPI-dense foci, in agreement with transcriptionally inactive sequences. This repressive environment is also established during conversion from ESCs to cEpiSCs *in vitro*, making it a relevant model to study the dynamics of these epigenetic and transcriptional changes. Although ChIP-qPCR suggests that a low level of H3K27me3 remains at PCH in primed EpiSCs, the pattern is only diffuse, with no prominent accumulation at chromocenters. In addition, PCH in primed EpiSCs seems refractory to increased PRC2 activity upon removal of H3K9me3 or 5-meC, as neither *Suv39h*dn nor *Dnmt*TKO converted cells gained H3K27me3 at their chromocenters. In this regard, EpiSCs resemble somatic cells such as MEFs^29^. We can also argue that in this somatic-like context, the presence of HP1α at PCH prevents further H3K27me3 enrichment^54^. It has been shown that DNMTs are recruited to H3K9me3-enriched PCH foci in ESCs, probably through interaction with SUV39H/HP1^30,55^. Therefore, DNA methylation is reduced at PCH in *Suv39h*dn serum-ESCs^30^. Our data show that in primed EpiSCs such interaction is not necessary, since *Suv39h*dn converted cells regain DNA methylation at PCH sequences. As DNMT1 has been shown to bind to PCH in a SUV39H independent manner^56^, it may be advantageous to maintain DNA methylation at PCH once established. Nevertheless, how this pattern of DNA methylation is established in converted EpiSCs in absence of SUV39H remains to be explored.

Finally, we observed that patterns of H3K9me3 and H3K27me3 in ESCs were particularly heterogeneous in the 2i culture condition. In addition, only one third of the cell population displayed local accumulation of satellite transcripts, as revealed by RNA-FISH. NANOG has been shown to play a role in reducing H3K9me3 deposition at PCH^57^, which is not consistent with its homogenous expression in 2i-ESCs (Supplementary Fig. 5) and the heterogeneity of this mark’s pattern. It is possible that the heterogeneity of H3K27me3/H3K9me3 patterns is a result of cell-to-cell transcriptome variability^9^. Alternatively, it could be due to globally relaxed chromatin allowing stochastic reshuffling of the repressive marks at each cell cycle. However, the proportions of different categories remained globally stable across passages, and this pattern seems to be an intrinsic property of any given ESC line. Indeed, the three different lines used in our study display variable proportions of these categories. We note that they differ in their genotype, as the ESC line used in Fig. 1 is from 129 backgrounds (R1) while the two others are from 129/B6 hybrids. It would also be interesting to assess whether such differences are linked to functional properties such as chimaera formation or chromosomal abnormalities.

In conclusion, the present study brings new insights into the organization of constitutive heterochromatin in naive and primed pluripotent cells *in vitro*. These findings have important implications in the definition of the naive state and set important landmarks to follow the efficiency of reprogramming towards this state. We believe that the *in vitro* conversion of naive to primed pluripotency will also allow us to decipher the molecular players controlling the epigenetic state of the PCH.

## Materials and Methods

### Cell culture

ESC lines were cultured in 2i or serum medium as described^58,59,60^. Briefly, 2i-ESCs were cultured on Laminin in Chemically Defined Medium (CDM)^2^ supplemented with LIF (700 U/ml), PD0332552 (1 µM) and CHIR99201 (final 3 µM), while serum-ESCs were cultured on gelatin in DMEM supplemented with 15% serum and LIF (1000 U/ml). *In vitro* conversion of ESC into cEpiSC was performed by switching ESCs from 2i/LIF medium to CDM supplemented with FGF2 (12 ng/ml) and Activin A (20 ng/ml) as described^61^. These converted cells were used 3–5 passages after the conversion, except for *Dnmt*TKO cells that were used after 10 days of conversion (P1). EpiSC lines (FT129.1 and 9.73) were cultured as described^2^. EZH2 inhibition was performed by culturing ESC either in 2i or serum-containing medium supplemented with 1 µM EPZ-6438 (AxonMedchem) for 72h (changing medium daily) or with DMSO as control. Wildtype ESCs were R1, WT01 (control for *Suv39hdn*) and 159-WT (control for *Dnmt*TKO). WT01 and *Suv39hdn* ESCs were obtained from A. Peters^30^ and 159-WT and *Dnmt*TKO were a gift from D. Schubeler^62^. A second *Dnmt*TKO ESC was also obtained from M. Okano^39^.

### Immunostaining

Cells were grown on coated glass-coverslips for 24 h, then fixed with PFA 2% (EMS) for 20 min, permeabilized with Triton X100 0.05% for 30 min and blocked with BSA 2% for 1 h. Primary antibody was incubated at 4 °C O/N. After washes, the secondary antibody was incubated for 1 h. Cells were then washed, post-fixed with PFA 2% (EMS) for 20  min, incubated with 1/500 DAPI (Invitrogen) at 37 °C for 15 min and finally mounted on slide with VectaShield (Vector Laboratories). Antibodies used are described in Supplementary Table S1.

### RNA-FISH

Cells grown on coated glass-coverslips for 24 h were fixed for 10 min in PFA 4%, rinsed in PBS and permeabilized in Triton X100 0.5% for 30 min. After washing with PBS and SSC 2x pH6.3, the coverslips were incubated O/N at 37 °C with the hybridation mix (50% formamide, 10% Dextran, 2x SSC) containing 1/100 tRNA and probes at 10 µM. After washing three times at 42 °C with SSC 2X pH6.3, cells were incubated in DAPI (Invitrogen) 1/500 at 37 °C 15min and mounted with VectaShield (Vector Laboratories). We used a mix of forward (MajF-5G-Alexa488) and reverse (majR-R4Y-Cy5) probed for detection, which were a generous gift from C. Escudé (MNHN).

### 3D-structured image acquisition and analysis

Imaging was performed at the MIMA2 platform (http://www6.jouy.inra.fr/mima2) with an inverted ZEISS AxioObserver Z1 microscope equipped with an ApoTome slider, a Colibri light source, Axiocam MRm camera and driven by the Axiovision software 4.8.2. Observations were carried out using a 63X oil-immersion objective. Cells were scanned entirely using a z-distance of 0.24 μm between optical sections. Fluorescent wavelengths of 405, 488, 555, and 639 nm were used to excite DAPI, FITC, Cy3, and Cy5, respectively. Images were then analysed on ImageJ (imagej.nih.gov/ij) to perform merge of channels and z-projections.

### Western blot

Cells were lysed for 30 min on ice into RIPA buffer (150 mM NaCl, 1% NP-40, 0.5% NaDeoxycholate, 0.1% SDS, 50mM Tris-HCl pH8.0) in presence of protease and phosphatase inhibitors (Pierce). Proteins were quantified using BCA assay (Pierce). 3 µg of proteins were charged on pre-cast polyacryalamide gel 4–15% (Biorad) for 1h run at 100V. Transfer was then performed on Trans-Blot Turbo (Biorad) for 7min on a PVDF membrane (Hybond-P, GE Healthcare). After transfer the membrane is divided into 3 parts, up (70 kD to>250 kD), middle (30–70 kD) and down (<30 kD) to allow simultaneous detection of antigens at different molecular weights. After blocking in TBS-Tween 20 0.01% (TBS-T) with either 4% non-fatty milk or 5% BSA, the membranes were incubated O/N at 4 °C with primary antibodies. After washes in TBS-T, the membranes were incubated with secondary antibodies for 1 h and washed again before the revelation with ECL2 Western blotting substrate (Pierce). Chemiluminescent signals were captured on a Fuji camera LAS-1000plus and then analysed with ImageJ (imagej.nih.gov/ij). For sequential protein detection, membranes were stripped with 25 mM Glycine and 1% SDS at pH 2 for 30 min, followed by washes in TBS-T and blocking (milk or BSA) according to the new primary antibody. Signals were normalized to H3 or Actin and data were presented relative to 2i-ESC (set as 1). Western-blots were repeated at least twice. Antibodies are described in Supplementary Table S1.

### DNA methylation analysis of satellite repeats using Southern blot

Southern blot on genomic DNA was performed as described in Thijssen *et al*.^63^. For major satellite analysis 200 ng of genomic DNA were digested with HpyCH4IV (New England Biolabs) for 1h at 37 °C, while for minor satellites 500ng of gDNA were digested with HpaII (New England Biolabs) and 300 ng with MspI (New England Biolabs), both O/N at 37 °C. Digested samples were separated for 5 h on 1% agarose gel. Gels were then denaturated in a 1.5 M NaCl and 0.5 M NaOH solution for 20 min and neutralized with 0.5 M Tris-HCl pH 7.5 and 1.5 M NaCl for 40 min. Transfer was performed O/N on Hybond-N+ membranes (GE Healthcare) in SSC 20X. After ultraviolet crosslinking, membranes were pre-hybridized in SSC 6X, Denhardt 5X and 0.1% SDS for 1 h at 42 °C and hybridized with 32P-labelled probes for 2 h at 42 °C. After membrane washing, signals were detected using FLA 7000 phosphorimager (Fuji). Images were then analyzed with ImageJ (imagej.nih.gov/ij) to perform linescan for major satellites and intensity ratio HpaII/MspI for the lower six bands of each lane for minor satellites. Probe used: Major satellites 5′-CAC GTC CTA CAG TGG ACA TTT CTA AAT TTT CCA CCT TTT TCA GTT-3′ and minor satellites 5′-ACA TTC GTT GGA AAC GGG ATT TGT AGA ACA GTG TAT ATC AAT GAG TTA CAA TGA GAA ACA T-3′.

### qRT-PCR

Total RNA was extracted from cells using TRIzol (Ambion). 3 µg of RNA were subjected to DNAse treatment using Turbo DNA-free kit (Ambion). Retrotranscription of 500 ng of DNAse treated-RNA was performed using Random primers (Invitrogen) and Superscript III (Invitrogen). For each sample, a negative control was included (no Superscript enzyme). Quantitative PCR was carried out in triplicates using SybrGreen mix (Applied Biosystem) on a StepOne Plus thermal cycler (Applied Biosystem). Data were normalized using the geometric mean of Sdha and Pbgd using Qbase software (Biogazelle). Results were presented according to Weissgerber *et al*.^64^. The primers are described in Supplementary Table S2.

### Chromatin immunoprecipitation

Cells were cross-linked directly in the plates with 1% formaldehyde for 5 mn, before quenching for 5 mn in 0.125 M glycine. After PBS rinsing, cells were pelleted and snap-frozen. Chromatin preparation and immune-precipitation was done using the True MicroChIP kit (Diagenode). Briefly, 100,000 cells resuspended in lysis buffer were sonicated in a Bioruptor (Diagenode), with 3 cycles of 30 s ON, 30s OFF. Fragmented chromatin was then incubated overnight with 1 µg of H3K27me3 or H3K9me3 antibody and recovered using magnetic beads according to the manufacturer’s protocol. After reverse-cross-linking for 4 hours at 65 °C, precipitated DNA as well as input were purified using MicroChIP DiaPure columns (Diagenode), diluted 100 times before being used as a template for q-PCR. Immunoprecipitation with 1 µg rabbit IgG was used as negative control and this never yields enrichment above 0.1%. Three to four independent replicates were used.

## Electronic supplementary material

Supplementary Figures &Tables

## References

[1] Nichols J, Smith A (2009). Naive and Primed Pluripotent States. Cell Stem Cell.

[2] Brons IGM (2007). Derivation of pluripotent epiblast stem cells from mammalian embryos. Nature.

[3] Tesar PJ (2007). New cell lines from mouse epiblast share defining features with human embryonic stem cells. Nature.

[4] Huang Y, Osorno R, Tsakiridis A, Wilson V (2012). *In Vivo* Differentiation Potential of Epiblast Stem Cells Revealed by Chimeric Embryo Formation. Cell Rep..

[5] Ying Q-L (2008). The ground state of embryonic stem cell self-renewal. Nature.

[6] Marks H (2012). The Transcriptional and Epigenomic Foundations of Ground State Pluripotency. Cell.

[7] Wray J, Kalkan T, Smith AG (2010). The ground state of pluripotency. Biochem. Soc. Trans..

[8] Chen, G. *et al*. Single-cell analyses of X Chromosome inactivation dynamics and pluripotency during differentiation. *Genome Res*. gr.201954.115 10.1101/gr.201954.115 (2016). 10.1101/gr.201954.115 PMC5052059 27486082

[9] Kolodziejczyk AA (2015). Single Cell RNA-Sequencing of Pluripotent States Unlocks Modular Transcriptional Variation. Cell Stem Cell.

[10] Ahmed K (2010). Global Chromatin Architecture Reflects Pluripotency and Lineage Commitment in the Early Mouse Embryo. PLoS ONE.

[11] Meshorer E (2006). Hyperdynamic Plasticity of Chromatin Proteins in Pluripotent Embryonic Stem Cells. Dev. Cell.

[12] Ricci MA, Manzo C, García-Parajo MF, Lakadamyali M, Cosma MP (2015). Chromatin Fibers Are Formed by Heterogeneous Groups of Nucleosomes *In Vivo*. Cell.

[13] Joshi O (2015). Dynamic Reorganization of Extremely Long-Range Promoter-Promoter Interactions between Two States of Pluripotency. Cell Stem Cell.

[14] Habibi E (2013). Whole-Genome Bisulfite Sequencing of Two Distinct Interconvertible DNA Methylomes of Mouse Embryonic Stem Cells. Cell Stem Cell.

[15] Guo G (2009). Klf4 reverts developmentally programmed restriction of ground state pluripotency. Development.

[16] Factor DC (2014). Epigenomic Comparison Reveals Activation of “Seed” Enhancers during Transition from Naive to Primed Pluripotency. Cell Stem Cell.

[17] Veillard, A.-C., Maruotti, J. & Jouneau, A. Reprogramming and pluripotency of epiblast stem cells. in *Stem cells and Cancer stem cells* (ed. Hayat, M. A.) **8**, 133–146 (Springer Science + Business Media, 2012).

[18] Ferreira D (2015). Satellite non-coding RNAs: the emerging players in cells, cellular pathways and cancer. Chromosome Res..

[19] Bao, S. *et al*. Epigenetic reversion of postimplantation epiblast cells to pluripotent embryonic stem cells. *Nature* **461** (2009). 10.1038/nature08534 PMC3863718 19816418

[20] Déjardin J (2015). Switching between Epigenetic States at Pericentromeric Heterochromatin. Trends Genet..

[21] Martens JH (2005). The profile of repeat‐associated histone lysine methylation states in the mouse epigenome. EMBO J..

[22] Peters AH (2003). Partitioning and plasticity of repressive histone methylation states in mammalian chromatin. Mol. Cell.

[23] Okano M, Bell DW, Haber DA, Li E (1999). DNA methyltransferases Dnmt3a and Dnmt3b are essential for de novo methylation and mammalian development. Cell.

[24] von Meyenn, F. *et al*. Impairment of DNA Methylation Maintenance Is the Main Cause of Global Demethylation in Naive Embryonic Stem Cells. *Mol*. *Cell* **0** (2016). 10.1016/j.molcel.2016.06.005 PMC4914604 27315559

[25] Lu J, Gilbert DM (2007). Proliferation-dependent and cell cycle–regulated transcription of mouse pericentric heterochromatin. J. Cell Biol..

[26] Camacho OV (2017). Major satellite repeat RNA stabilize heterochromatin retention of Suv39h enzymes by RNA-nucleosome association and RNA:DNA hybrid formation. eLife.

[27] Eymery A, Callanan M, Vourc’h C (2009). The secret message of heterochromatin: new insights into the mechanisms and function of centromeric and pericentric repeat sequence transcription. Int. J. Dev. Biol..

[28] Efroni S (2008). Global Transcription in Pluripotent Embryonic Stem Cells. Cell Stem Cell.

[29] Cooper S (2014). Targeting Polycomb to Pericentric Heterochromatin in Embryonic Stem Cells Reveals a Role for H2AK119u1 in PRC2 Recruitment. Cell Rep..

[30] Lehnertz B (2003). Suv39h-mediated histone H3 lysine 9 methylation directs DNA methylation to major satellite repeats at pericentric heterochromatin. Curr. Biol..

[31] Bulut-Karslioglu A (2014). Suv39h-Dependent H3K9me3 Marks Intact Retrotransposons and Silences LINE Elements in Mouse Embryonic Stem Cells. Mol. Cell.

[32] Saksouk N (2014). Redundant Mechanisms to Form Silent Chromatin at Pericentromeric Regions Rely on BEND3 and DNA Methylation. Mol. Cell.

[33] Margueron R, Reinberg D (2011). The Polycomb complex PRC2 and its mark in life. Nature.

[34] Chen T, Ueda Y, Xie S, Li E (2002). A Novel Dnmt3a Isoform Produced from an Alternative Promoter Localizes to Euchromatin and Its Expression Correlates with Activede Novo Methylation. J. Biol. Chem..

[35] Knutson SK (2013). Durable tumor regression in genetically altered malignant rhabdoid tumors by inhibition of methyltransferase EZH2. Proc. Natl. Acad. Sci..

[36] Knutson SK (2014). Selective Inhibition of EZH2 by EPZ-6438 Leads to Potent Antitumor Activity in EZH2-Mutant Non-Hodgkin Lymphoma. Mol. Cancer Ther..

[37] Mozzetta C, Boyarchuk E, Pontis J, Ait-Si-Ali S (2015). Sound of silence: the properties and functions of repressive Lys methyltransferases. Nat. Rev. Mol. Cell Biol..

[38] Schultz DC, Ayyanathan K, Negorev D, Maul GG, Rauscher FJ (2002). SETDB1: a novel KAP-1-associated histone H3, lysine 9-specific methyltransferase that contributes to HP1-mediated silencing of euchromatic genes by KRAB zinc-finger proteins. Genes Dev..

[39] Tsumura A (2006). Maintenance of self-renewal ability of mouse embryonic stem cells in the absence of DNA methyltransferases Dnmt1, Dnmt3a and Dnmt3b. Genes Cells.

[40] Schmidt CS (2012). Global DNA Hypomethylation Prevents Consolidation of Differentiation Programs and Allows Reversion to the Embryonic Stem Cell State. PLOS ONE.

[41] Osorno R (2012). The developmental dismantling of pluripotency is reversed by ectopic Oct4 expression. Development.

[42] Casanova M (2013). Heterochromatin Reorganization during Early Mouse Development Requires a Single-Stranded Noncoding Transcript. Cell Rep..

[43] Maison C (2002). Higher-order structure in pericentric heterochromatin involves a distinct pattern of histone modification and an RNA component. Nat. Genet..

[44] Bouzinba-Segard H, Guais A, Francastel C (2006). Accumulation of small murine minor satellite transcripts leads to impaired centromeric architecture and function. Proc. Natl. Acad. Sci..

[45] Hagarman JA, Motley MP, Kristjansdottir K, Soloway PD (2013). Coordinate Regulation of DNA Methylation and H3K27me3 in Mouse Embryonic Stem Cells. PLOS ONE.

[46] ter Huurne M, Chappell J, Dalton S, Stunnenberg HG (2017). Distinct Cell-Cycle Control in Two Different States of Mouse Pluripotency. Cell Stem Cell.

[47] Bulut-Karslioglu A (2012). A transcription factor–based mechanism for mouse heterochromatin formation. Nat. Struct. Mol. Biol..

[48] Illingworth RS, Hölzenspies JJ, Roske FV, Bickmore WA, Brickman JM (2016). Polycomb enables primitive endoderm lineage priming in embryonic stem cells. eLife.

[49] Liao, J. *et al*. Targeted disruption of DNMT1, DNMT3A and DNMT3B in human embryonic stem cells. *Nat*. *Genet*. advance online publication (2015). 10.1038/ng.3258 PMC4414868 25822089

[50] Peters AHFM (2001). Loss of the Suv39h Histone Methyltransferases Impairs Mammalian Heterochromatin and Genome Stability. Cell.

[51] Krouwels IM (2005). A glue for heterochromatin maintenance: stable SUV39H1 binding to heterochromatin is reinforced by the SET domain. J. Cell Biol..

[52] Müller‐Ott K (2014). Specificity, propagation, and memory of pericentric heterochromatin. Mol. Syst. Biol..

[53] Sim Y-J (2017). 2i Maintains a Naive Ground State in ESCs through Two Distinct Epigenetic Mechanisms. Stem Cell Rep..

[54] Bosch-Presegué L (2017). Mammalian HP1 Isoforms Have Specific Roles in Heterochromatin Structure and Organization. Cell Rep..

[55] Fuks F, Hurd PJ, Deplus R, Kouzarides T (2003). The DNA methyltransferases associate with HP1 and the SUV39H1 histone methyltransferase. Nucleic Acids Res..

[56] Easwaran HP, Schermelleh L, Leonhardt H, Cardoso MC (2004). Replication‐independent chromatin loading of Dnmt1 during G2 and M phases. EMBO Rep..

[57] Novo, C. L. *et al*. The pluripotency factor Nanog regulates pericentromeric heterochromatin organization in mouse embryonic stem cells. *Genes Dev*. 10.1101/gad.275685.115 (2016). 10.1101/gad.275685.115 PMC4863740 27125671

[58] Tosolini M, Jouneau A (2016). Acquiring Ground State Pluripotency: Switching Mouse Embryonic Stem Cells from Serum/LIF Medium to 2i/LIF Medium. *Methods Mol*. Biol. Clifton NJ.

[59] Hassani S-N (2014). Inhibition of TGFβ Signaling Promotes Ground State Pluripotency. Stem Cell Rev. Rep..

[60] Choi J (2017). Prolonged Mek1/2 suppression impairs the developmental potential of embryonic stem cells. Nature.

[61] Tosolini M, Jouneau A (2016). From Naive to Primed Pluripotency: *In Vitro* Conversion of Mouse Embryonic Stem Cells in Epiblast Stem Cells. *Methods Mol*. Biol. Clifton NJ.

[62] Domcke S (2015). Competition between DNA methylation and transcription factors determines binding of NRF1. Nature.

[63] Thijssen PE (2015). Mutations in CDCA7 and HELLS cause immunodeficiency-centromeric instability-facial anomalies syndrome. Nat. Commun..

[64] Weissgerber TL, Milic NM, Winham SJ, Garovic VD (2015). Beyond bar and line graphs: time for a new data presentation paradigm. PLoS Biol..

