# Supplementary material for: Contrasting epigenetic states of heterochromatin in the different types of mouse pluripotent stem cells
Source: Sci Rep. 2018 Apr 10;8:5776. doi: 10.1038/s41598-018-23822-4 (PMC5893598; doi:10.1038/s41598-018-23822-4)
Supplement: Supplementary file 1 — Supplementary Figures &Tables [file 41598_2018_23822_MOESM1_ESM.pdf]

Supplementary Figures and Tables

Contrasting epigenetic states of heterochromatin in the different types of mouse pluripotent stem cells

Tosolini Matteo, Brochard Vincent, Adenot Pierre, Chebrout Martine, Grillo Giacomo, Navia Violette, Beaujean Nathalie, Francastel Claire, Bonnet-Garnier Amélie and Jouneau Alice

Supplementary Figure legends

Supplementary Fig. 1:

(A) Magnification of nuclei of 2i-ESCs, serum-ESCs and EpiSCs, representative of the categories described in Fig. 1B. (B) Mean number of H3K9me3, H3K27me3 and DAPI clusters in each category and each type of pluripotent cells.

Supplementary Fig.2

RNA-FISH for Major satellites in ESCs: examples of nuclei showing localization of RNA foci near chromocenter (A), within peri-nuclear heterochromatin (B) or peri-nucleolar heterochromatin (C). DAPi is in red, major satellite probe is in green.

Supplementary Fig. 3:

(A) Pictures of EpiSCs converted from WT or Suv39hdn ESCs. (B) Relative expression of different common pluripotency (*Oct4, Sox2, Nanog*), naive-specific (*Klf4, Esrrb*) and epiblast specific (*Dnmt3b*, *Fgf5, Otx2*) transcripts by qRT-PCR analysis normalized to *Sdha* and *Pbgd* housekeeping genes. 2i-ESC expression was set to 1.Three independent conversions were made. (C) Western-blot analysis of EZH2, H3K27me3 and H3K9me3 and H3 as loading control, in WT and Suv39hdn cells. (D) Immunostaining images (single plan) for H3K9me3, H3K27me3 and DAPI in WT 2i-ESCs and WT cEpiSCs. (E) Representative nuclei of the categories as in Fig. 1B and percentages of each in the WT 2i-ESC (WT01 line) and cEpiSC populations. (F) ChIP-QPCR analysis of H3K9me3 and H3K27me3 at major satellites in WT and *Suv39hdn* 2i-ESCs and cEpiSCs (n=4; error bars are s.e.m). Values were normalized to input and shown relative to WT 2i-ESCs.

Supplementary Fig. 4:

(A) Pictures of ESCs and cEpiSCs obtained after 10 days of conversion. (B) Relative expression of different common pluripotency (*Oct4*, *Sox2*, *Nanog*), naive-specific (*Klf4*, *Esrrb*) and epiblast specific (*Fgf5*, *Otx2*) transcripts by qRT-PCR analysis normalized to *Sdha* and *Pbgd* housekeeping genes. 2i-ESC expression was set to 1. Two independent conversions were made. (C) Percentages of each category of nuclei for WT-ESCs and WT cEpisCs.

Supplementary Fig. 5

Immunostaining images of NANOG in the 3 pluripotent cell types.

Supplementary Table S1: List of antibodies used for immunostaining and western-blot

Supplementary Table S2: list of primers used for qRT-PCR

A

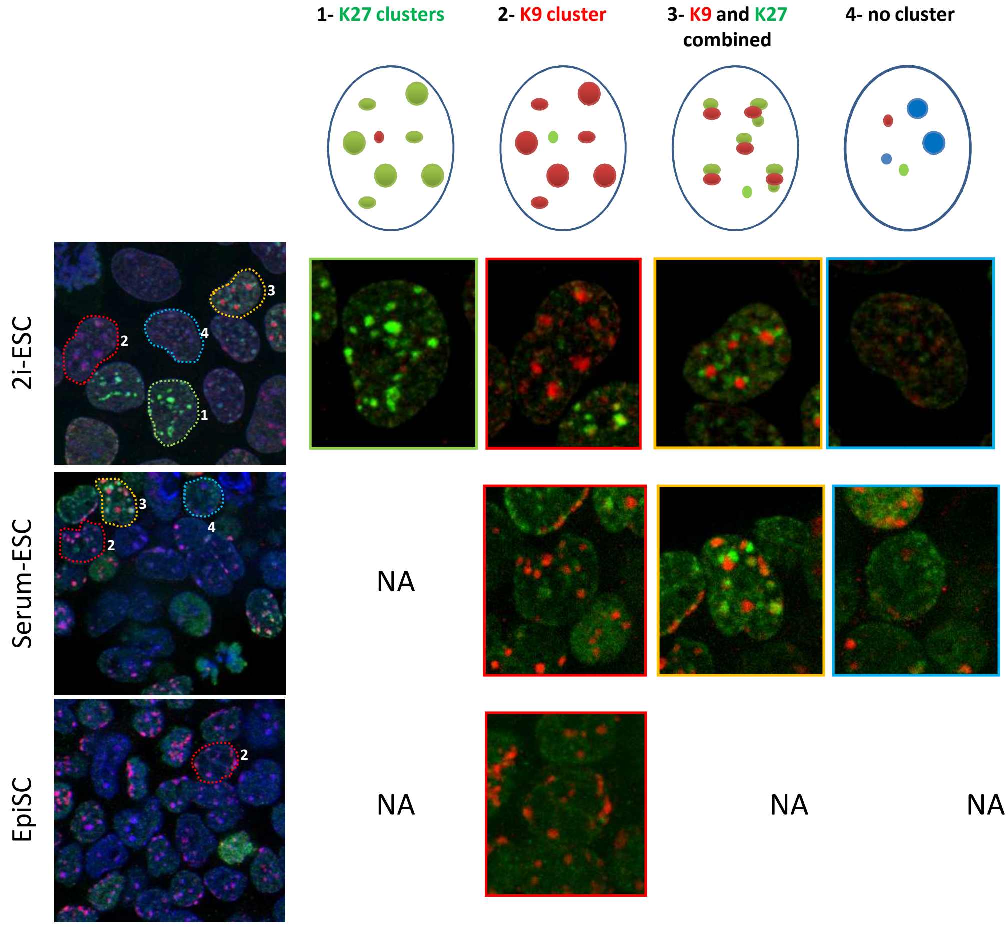

B

Mean number of clusters/ categories	Cat. 1	Cat. 2	Cat. 3	Cat. 4
2i-ESC (n=75)				
**DAPI**	**17**	**14**	**11**	**15**
K9me3	2	11	9	2
K27me3	17	2	9	1
serum ESC (n=40)				
**DAPI**	**NA**	**16**	**16**	**7**
K9me3	NA	18	13	2
K27me3	NA	1	7	1
EpiSC (n=30)				
**DAPI**	**NA**	**15**	**NA**	**NA**
K9me3	NA	15	NA	NA
K27me3	NA	0	NA	NA

Supp Figure 1

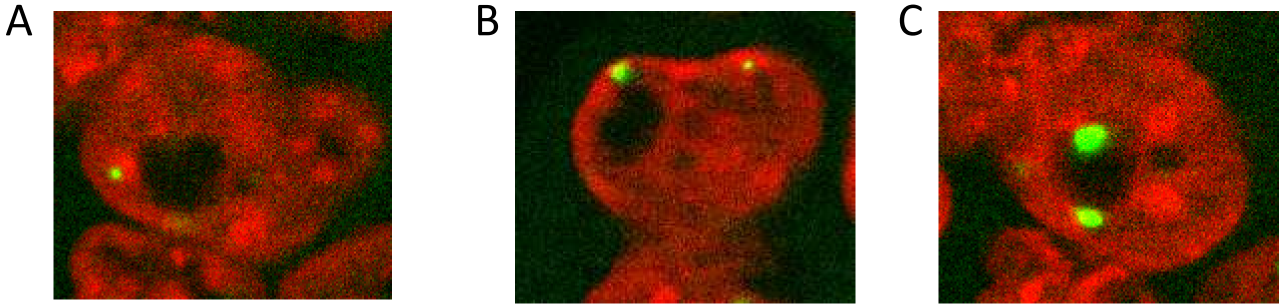

Supplementary Fig.2
RNA-FISH for Major satellites in ESCs: examples of nuclei showing localization of RNA foci near chromocenter (A), within peri-nuclear heterochromatin (B) or peri-nucleolar heterochromatin (C).

Supp Figure 2

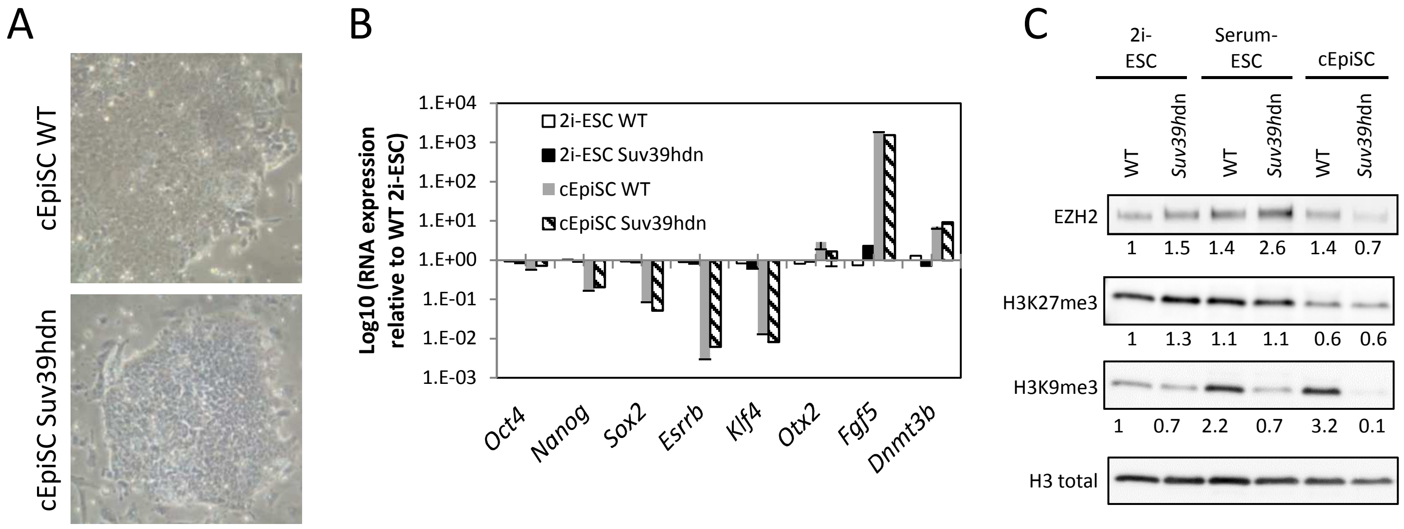

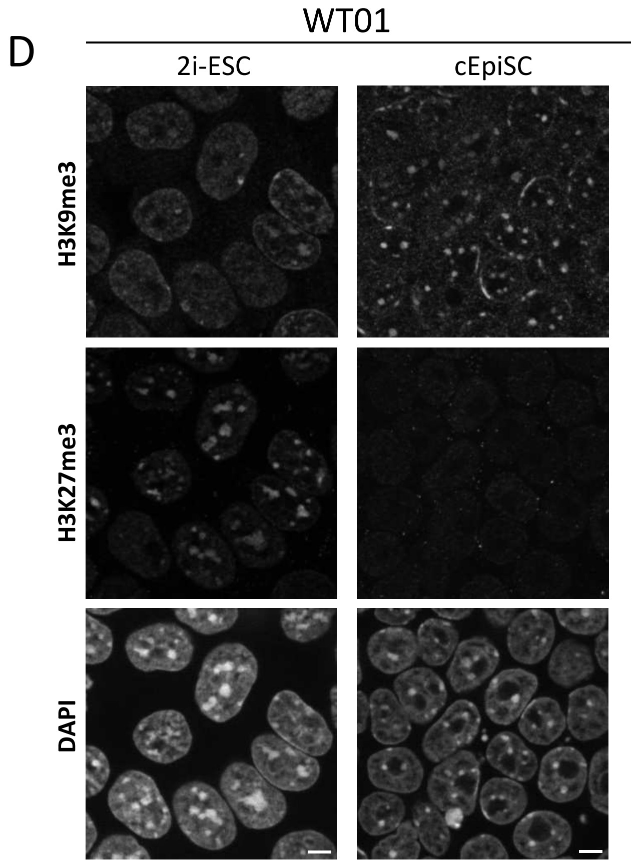

E

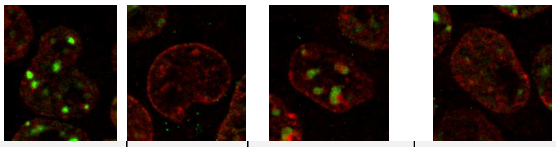

Category of nucleus	1- K27 cluster	2- K9 cluster	3- K9 and K27 combined	4- No cluster
WT01 2i-ESCs (n=49)	67%	4%	14%	14%
WT01 cEpiSC (n=20)	0%	100%	0%	0%

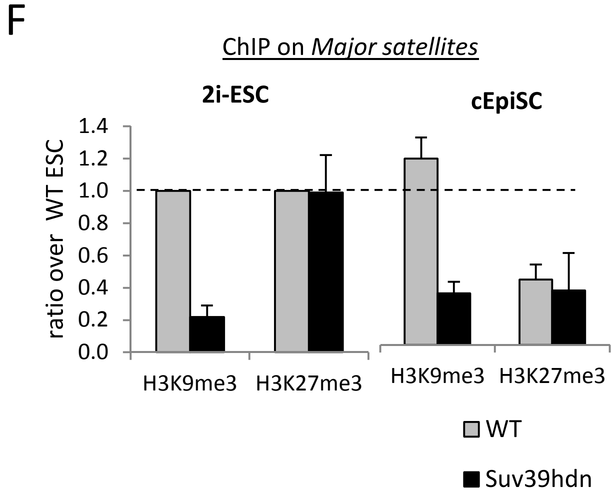

Supp Figure 3

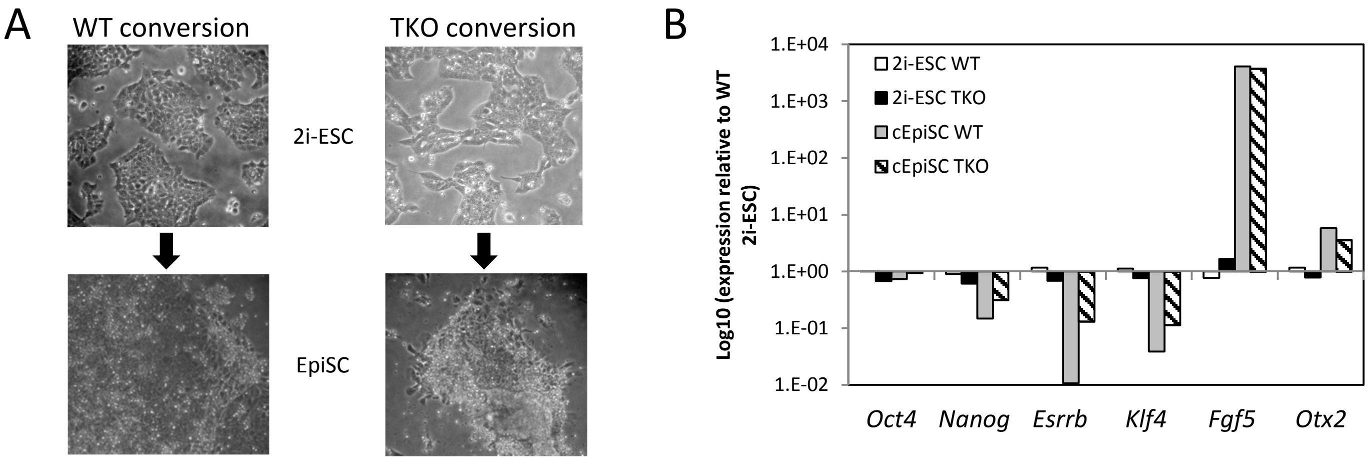

C

Category of nucleus	1- K27 cluster	2- K9 cluster	3- K9 and K27 combined	4- No cluster
159-WT 2i-ESCs (n=30)	40%	21%	40%	0%
159-WT EpiSC (n=17)	0%	100%	0%	0%

Supp Figure 4

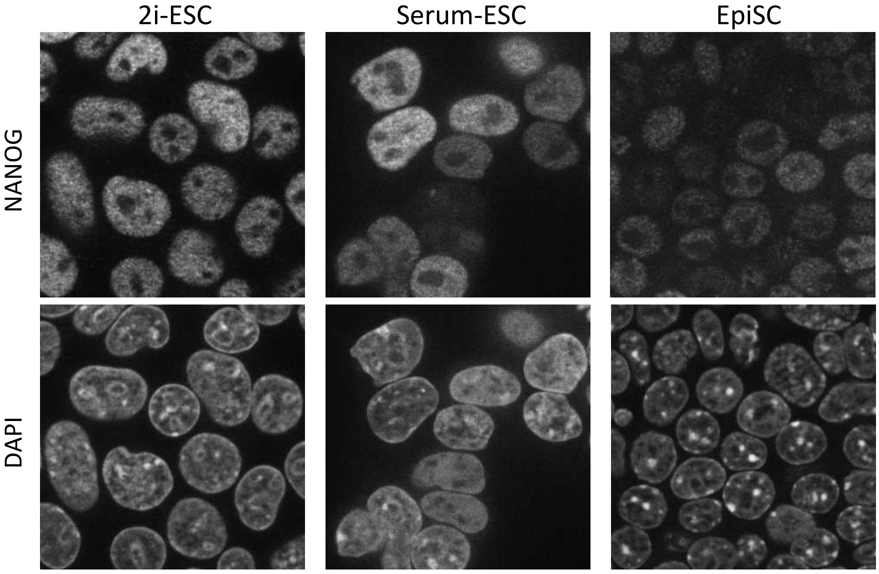

Supp Figure 5

Antigen	dilution for immunostaining	dilution for western-blot	reference
H3K9me3	1/300	1/1000	Active Motif 39161
H3K27me2me3	1/300	1/1000	Active Motif 39538
H3K27me3		1/1000	Millipore DAM07-774
EZH2	1/200	1/1000	Novocastra NCL-L-EZH
EZH1		1/500	Active Motif 61583
SUV39H1	1/100	1/1000	Cell Signaling D11B6 #8729
NANOG	1/100		Abcam ab80892
DNMT3B	1/200	1/500	Active Motif 39207
DNMT3A		1/500	Active Motif 39206
H3total		1/20,000	Abcam 1791
Anti-Rabbit-Cy3	1/200		Jackson ImmunoResearch
Anti-Mouse-FITC	1/200		Jackson ImmunoResearch
Anti-Goat-Cy3	1/200		Jackson ImmunoResearch
Anti-mouse-HPO		1/5000	Jackson ImmunoResearch
anti-rabbit-HPO		1/5000	Jackson ImmunoResearch

Supp Table S1

Primers for qRT-PCR				
**Gene**	**Primer Forward Sequence (5'-3')**	**Primer Revers Sequence (5'-3')**	**Reference or Primer Bank ID**	**Annealing**
Major satellite	GACGACTTGAAAAATGACGAAATC	CATATTCCAGGTCCTTCAGTGTGC	1	60°C
Minor satellite	GAACATATTAGATGAGTGAGTTAC	GTTCTACAAATCCCGTTTCCAAC	2	60°C
b-Actin promoter	CGTATTAGGTCCATCTTG	GCCATTGAGGCGTGATC		60°C
Sdha	GGAACACTCCAAAAACAGACCT	CCACCACTGGGTATTGAGTAGAA		60°C
Pbgd	CCTGGCATACAGTTTGAAATCAT	TTTTTCCAGGGCGTTTTCT	3	60°C
Ezh2	AGTGACTTGGATTTTCCAGCAC	AATTCTGTTGTAAGGGCGACC		60°C
Suv39h1	GCAGTGTGTGCTGTAAATCTTCT	ATACCCACGCCACTTAACCAG		60°C
Dnmt3a	GAGGGAACTGAGACCCCAC	CTGGAAGGTGAGTCTTGGCA	6681209a1	60°C
Dnmt3b	TCAGATGAGCAAGGTCAAGG	TGTACCAAAGCAAGGGGAAG		60°C
Oct4	CAGCCAGACCACCATCTGTC	GTCTCCGATTTGCATATC	7305399a3	58°C
Nanog	CTTTCACCTATTAAGGTGCTTGC	TGGCATCGGTTCATCATGGTAC	4	58°C
Sox2	GCGGAGTGGAAACTTTTGTCC	CGGGAAGCGTGTACTTATCCTT		60°C
Fgf5	TGTGTCTCAGGGGATTGTAGG	AGCTGTTTTCTTGGAATCTCTCC	6753854a1	60 °C
Otx2	TATCTAAAGCAACCGCCTTACG	GCCCTAGTAAATGTCGTCCTCTC	158518427c1	60°C
Esrrb	ATGCGAGTACATGCTTAACGC	CATCCCCACTTTGAGGCATTT		60°C
Klf4	GCAGTCACAAGTCCCCTCTC	GACCTTCTTCCCCTCTTTGG	5	58°C

1. Lehnertz, B. et al. Suv39h-mediated histone H3 lysine 9 methylation directs DNA methylation to major satellite repeats at pericentric heterochromatin. Curr. Biol. 13, 1192–1200 (2003).
2. Ferri, F., Bouzinba-Segard, H., Velasco, G., Hubé, F. & Francastel, C. Non-coding murine centromeric transcripts associate with and potentiate Aurora B kinase. Nucleic Acids Res. 37, 5071–5080 (2009).
3. Bernardo, A. S. et al. BRACHYURY and CDX2 Mediate BMP-Induced Differentiation of Human and Mouse Pluripotent Stem Cells into Embryonic and Extraembryonic Lineages. Cell Stem Cell 9, 144–155 (2011).
4. Hayashi, K. & Surani, M. A. Self-renewing epiblast stem cells exhibit continual delineation of germ cells with epigenetic reprogramming in vitro. Development 136, 3549–3556 (2009).
5. Jouneau, A. et al. Naive and primed murine pluripotent stem cells have distinct miRNA expression profiles. RNA 18, 253–64 (2012).

Supp Table S2
